# Vitreous Analysis in the Management of Uveitis

**DOI:** 10.1155/2012/863418

**Published:** 2012-10-24

**Authors:** Erika M. Damato, Martina Angi, Mario R. Romano, Francesco Semeraro, Ciro Costagliola

**Affiliations:** ^1^Regional Ocular Inflammatory Service, Bristol Eye Hospital, Bristol BS1 2LX, UK; ^2^Department of Molecular and Clinical Cancer Medicine, University of Liverpool, Liverpool L69 3BX, UK; ^3^Department of Ophthalmology, Istituto Clinico Humanitas, Milan, Italy; ^4^Department of Ophthalmology, Spedali Civili di Brscia, University of Brescia, Brescia, Italy; ^5^Department of Health Sciences, University of Molise, Campobasso, Italy

## Abstract

A correct diagnosis of uveitis is often challenging, given the wide range of possible underlying conditions and the lack of typical phenotypes. Management decisions may be difficult in view of the risk of visual loss with either inappropriate or delayed therapy. Analysis of the vitreous may therefore be used to provide the clinician with valuable information. In this paper, we describe the main clinical situations in which vitreous sampling is indicated and provide some guidance to clinicians for tailoring their requests. These situations include suspected intraocular infection and suspected intraocular malignancy. We describe the principal tests carried out on vitreous samples, including cultures, polymerase chain reaction-based testing, and cytokine analysis. Limitations of the tests used are likely to become less as more advanced testing methods are introduced. The importance of selecting the appropriate investigations to support a clinical suspicion is emphasised, as is the interpretation of test results within a clinical context.

## 1. Introduction

The term “uveitis” encompasses a wide spectrum of conditions resulting in intraocular inflammation. Standardised uveitis nomenclature (SUN) defines uveitis depending on the predominant site of inflammation within the eye [1]. At the most severe end of the spectrum, uncontrolled or inadequately treated posterior uveitis may result in irreversible visual loss.

Uveitis may be associated with an underlying systemic disease or may exclusively involve the eye [2]. There are a vast number of causes and conditions related to the development of uveitis; however, these may be broadly divided into infectious, autoinflammatory, and neoplastic causes. Extensive investigations are often carried out to establish one, as the clinical phenotype may not be specific for a diagnosis. Common investigations include angiography, blood tests, urinalysis, chest X-rays, and CT scans. In certain situations, incorrect treatment may be catastrophic for vision and could potentially threaten the patient's life [3]. Hence, a rapid and accurate diagnosis based on intraocular sampling may be essential, mainly to exclude infection or malignancy before the introduction of powerful immunosuppressive or steroid therapy. The vitreous gel is amenable to sampling, either by vitreous tap, where a small amount of gel is aspirated with a needle or by a formal vitrectomy, where most or all of the vitreous gel is removed surgically [4].

In this paper, we describe the clinical situations in which vitreous sampling may become necessary, providing a guide to clinicians for tailoring their requests for laboratory analyses. We also review the salient immunological findings in the setting of experimental autoimmune uveitis and clinical studies, which may become relevant in future clinical practice. 

### 1.1. Sampling Intraocular Fluids

Analysis of a small sample of aqueous humour may be adequate in order to confirm a clinically suspected intraocular infection, in particular in the context of suspected viral retinitis [5].

Anterior chamber paracentesis has the advantage of being quick, relatively straightforward to perform and can be carried out in the outpatient setting [6]. Main limitations are that: (1) only about 0.2 mL of fluid are obtained, which may only be sufficient for one molecular test and (2) if there is relatively mild inflammation at the anterior part of the eye, then a false negative result may occur [5].

In order to obtain a larger sample (0.5 mL–1 mL) of intraocular fluid, vitreous sampling is necessary. This can be obtained by either a vitreous cutter or by using a 23 G needle. Formal pars plana vitrectomy requires an operation and needs to be carried out by a skilled ophthalmic surgeon. This allows up to 2 mL of undiluted vitreous to be sampled and sent for analysis. 

### 1.2. What Are the Indications for Sampling the Vitreous?

The three main indications for sampling the vitreous that will be described are

1. suspected intraocular infection;
2. suspected intraocular lymphoma;
3. atypical response to therapy during the treatment of presumed autoimmune intraocular inflammation.

## 2. Suspected Intraocular Infections

The two most common vision threatening intraocular infections are viral retinitis and infectious bacterial endophthalmitis. In both situations, irreversible visual loss can occur rapidly. Immediate therapy is warranted with appropriate antimicrobial agents, and often the clinical phenotype and clinical history will strongly direct the clinician toward a diagnosis. Vitreous sampling helps to confirm the clinical suspicion and to identify a causative agent, which has therapeutic implications. 

Tests carried out on the vitreous include cytological examination, culture of suspected organisms, and molecular analyses [7]. In clinical ophthalmology, the introduction of molecular diagnostics, mainly based on the polymerase chain reaction (PCR), has changed the management of patients, as it enables a rapid and tailored therapy [8]. PCR is more sensitive than culture for the detection of many organisms, and by utilizing the two together increased specificity can be assured [9]. PCR may; however, be affected by contaminants or by sample degradation, resulting in false positive results. Also there is a limit of detection below which the pathogen cannot be detected reliably, thus resulting in a false negative result. PCR techniques have been developed since their introduction, evolving from qualitative (presence/absence of pathogen) to quantitative or real-time PCR. Quantitative PCR is particularly useful in the clinical setting because it measures the pathogen (especially viral) load, hence allows monitoring of response to treatment over consecutive samples [10]. Multiplex PCR has also been introduced, which runs several primers at once, thus allowing several organisms to be detected and quantified simultaneously. 

### 2.1. Viral Retinitis

The classic clinical phenotype of viral retinitis is a rapidly progressive, confluent retinitis associated with a dense vitritis, anterior chamber activity, and raised intraocular pressure. The most common causative agents are the herpetic viruses including varicella zoster (VZV) and herpes simplex (HSV) [11]. The clinical picture may be pathognomonic, in which case empirical therapy with intravitreal and systemic antiviral agents is commenced independent of a PCR-based test. In some circumstances, however, the diagnostic certainty is less [12]. Other viruses including cytomegalovirus (CMV) and possibly Epstein-Barr virus (EBV) can also be involved [13]. In immunocompromised patients, the clinical phenotype may be consistent or concurrent with other infections, which must be excluded, including syphilis and toxoplasmosis [14]. In addition, the clinical picture in such patients is less extensive that what may be expected. Therefore, when a vitreous sample is sent from a patient with suspected infectious retinitis, PCR testing for HSV, VZV, CMV, EBV, and toxoplasmosis is often requested. 

Importantly, the presence of viral antigen, detected by qualitative PCR may not always be clinically relevant, for example, in viruses which can remain latent in host cells may be detected by PCR without actually being the cause of the retinitis. This is especially the case for testing EBV and CMV. 

The sensitivity and specificity of PCR testing in the context of viral retinitis has been investigated both on aqueous and vitreous humour specimens. For instance, Harper et al. reported that out of 113 patients, using the final clinical diagnosis as the gold standard, a true positive result was obtained by PCR in 76 patients, whilst a true negative result was obtained in 38. There were one false positive result and 18 false negative results [15]. The result may have been influenced by the use of anterior chamber paracentesis. Other authors have reported that vitreous sampling enables a much higher sensitivity for demonstrating viral PCR as compared with anterior chamber tap [16].

The likelihood of dual pathology is higher in immunocompromised patients, as these patients are more predisposed to developing retinitis in the first place. In such patients, vitreous sampling is preferred over aqueous sampling, as volumes for testing are greater. As mentioned previously, more than one positive PCR result may, however, simply reflect the “detection” of a “latent” infection, for example, in the case of EBV. In one study by Cochrane et al., more than one infectious agent was demonstrated in 12 out of 77 patients [16].

### 2.2. Bacterial and Fungal Endophthalmitis

Intraocular infection can occur secondary to bacteria, either introduced into the eye during surgery or from another source in the body travelling to the eye from the systemic circulation [17]. Intraocular bacterial endophthalmitis can be devastating for vision. Classically, postoperative infection presents as a painful red eye with a hypopyon and significant vitritis in the week following intraocular surgery. In this situation, a vitreous biopsy is taken and broad-spectrum antibiotics such as vancomycin and ceftazidime are introduced into the vitreous cavity while waiting for the laboratory results. In the laboratory, part of the vitreous specimen is put into culture, while the remainder is examined following immunohistochemical staining [18]. The most common responsible organisms identified in this setting include *Staphylococcus aureus*, *Staphylococcus epidermidis*, and *Streptococcus* species. 

Sensitivity can be significantly improved by the use of PCR, where correlation with culture results is high. A recent paper evaluated the efficacy of quantitative real-time PCR in the diagnosis of postoperative bacterial endophthalmitis among 64 patients who underwent cataract surgery. PCR allowed the detection of bacterial DNA in 66% of patients, compared to 34% with traditional culture. Only one patient had a positive result by culture (*Nocardia* species) but negative result by PCR [19].

Bispo et al. have analysed aqueous and vitreous taken from 14 eyes with suspected bacterial endophthalmitis. Gram staining and culture were followed by PCR testing of ocular fluids looking for bacterial infection. Testing was carried out for 31 clinically prevalent bacteria, including both gram positive and gram negative organisms. It was possible to perform gram staining in all samples; however, culture was only successful in just under half of patients. The use of PCR enabled a positive result to be obtained in 95% of patients. In this study the rate of false positive PCR results was low, occurring in 3.4% of patients [20]. 

Endogenous endophthalmitis refers to infection occurring within the eye as a consequence of systemic infection. In this situation, the patient is often clinically unwell or septic. The spectrum of causative organisms is different, and fungal infections are more relevant [21]. Patients also require systemic investigations, such as blood cultures, cardiac echocardiogram, or urine cultures. Systemic antimicrobial treatment also plays a major role. In a paper by Schiedler et al. [21], fungal infections such as *Candida* and *Aspergillus* were most commonly demonstrated on culture. Targeting the investigation to the clinical context increases the diagnostic yield, as a significant proportion of patients also had demonstrable fungaemia on blood culture. 

### 2.3. Chronic Postoperative Bacterial Endophthalmitis

Following intraocular surgery, such as phacoemulsification and intraocular lens insertion, some patients may be affected by chronic low-grade inflammation. It has been shown that it can be due to a low-grade infection caused by fastidious organisms such as *Propionibacterium acnes*, *Actinomyces israelii*, or *Corynebacterium spp.* [22]. Demonstration of such organisms by culture and microscopy is difficult, and sensitivities are low. This is attributable to the fact that these organisms may be present in very low numbers and grow very slowly in culture. A positive diagnosis of such fastidious organisms is greatly enhanced by the use of PCR. In a study investigating the delayed onset endophthalmitis following cataract surgery, vitreous testing allowed a causative organism to be identified in 92% of eyes, compared with 6% of eye using culture. In this setting, testing is guided by clinical suspicion, as the clinical phenotype may closely mimic idiopathic intraocular inflammation. Identification of an infectious cause will enable decisions regarding future therapy, such as surgical intervention and antimicrobial medication. The optimal management is controversial and strategies include systemic antibiotics, intracapsular antibiotics, and surgical removal of the intraocular lens, lens capsule, and vitreous. 

As well as the fastidious bacteria mentioned, mycobacterial species or fungi may be the cause of chronic post operative or delayed onset endophthalmitis. These organisms are also demonstrable by PCR, however, require a degree of clinical suspicion for these diagnoses to be considered. 

### 2.4. Toxoplasmosis

Toxoplasmosis is parasitic protozoan infection that can infect the retina either in utero or as a primary infection resulting in a characteristic chorioretinal scar. At times, the parasite may become reactivated, resulting in intraocular inflammation and evidence of activity or fluffy white areas around the scar. There is often vitritis and perivascular change associated with reactivation. The clinical phenotype is often typical, and usually treatment with the appropriate antimicrobials is commenced based on fundoscopic findings. 

In some patients, however, especially in patients who are immunocompromised, intraocular toxoplasmosis can result in a clinical phenotype very similar to acute retinal necrosis. In such cases, accurate diagnosis is imperative as incorrect therapy with antiviral therapy will not be effective, and retinitis may rapidly progress. Patients with ocular toxoplasmosis usually have antibodies (IgG) circulating in peripheral blood, and a negative serology may often be used to exclude the diagnosis. The most accurate diagnostic testing, however, is by using intraocular fluid [23].

Ocular fluids can be tested for local antibody production or for the presence of microbial DNA using PCR. Local antibody production can be detected using immunoblotting techniques, and a Goldmann-Witmer coefficient can be calculated to compare intraocular antibody production with serum antibody levels. A ratio of greater than 1.0 is abnormal and ratios of 2-3 are significant. 

In immunocompromised patients, however, antibody production is impaired, and molecular diagnostic plays an important role. In a study of 15 patients in whom a clinical diagnosis was unclear, PCR for toxoplasmosis enabled a diagnosis in 7. The remaining patients were diagnosed as having alternative conditions following further testing. In this paper, a volume of 0.4 mL of vitreous was used and qualitative PCR was utilised [24].

The use of both tests together increases the sensitivity of diagnosing toxoplasmosis as both may be affected by the immune status of the individual and by the stage of the disease. Toxoplasma DNA may not be detected until 2-3 weeks after the initiation of infection, therefore early testing may fail to demonstrate the organism, leading to a false negative result. Antibody testing is more likely to be positive in the early stages of infection and may also be affected by the use of steroids, commonly used in association with antibiotics, to treat the inflammatory component of the reactivation. This is supported by more than one study investigating the combined use of PCR and Goldmann-Witmer coefficient, noting, however, that this was carried out using aqueous humour samples [25, 26].

### 2.5. Intraocular Tuberculosis (TB)

Tuberculosis is implicated in intraocular inflammation either causing direct infection of intraocular tissues, where TB can be demonstrated within the eye, or resulting in immune-mediated inflammation affecting intraocular tissues. In the latter case, the presumption is that the presence of TB outside the eye results in intraocular inflammation due to an immune-mediated attack on intraocular tissues, presumably due to mimicry between TB antigen and retinal antigens. There has been significant interest in the use of interferon release assays such as QuantiFERON-Gold testing of blood in patients with presumed idiopathic uveitis or retinal vasculitis. 

Testing ocular fluids using PCR to detect TB is not routinely employed in UK uveitis clinics; however, this can be carried out. A larger volume of vitreous is required, compared to the amount required in testing for herpetic viruses. 

In countries where tuberculosis is more common than that in the UK, patients with uveitis demonstrating consistent clinical features, such as choroidal granulomas and retinal vasculitis, are often empirically treated with antituberculous therapy with good results. There is evidence to suggest that “idiopathic” retinal vasculitis, where there is clinical evidence of inflammation around blood vessels in the retina, or patients with presumed “Eales disease,” may actually have TB demonstrable inside the eye as demonstrated by PCR testing of vitreous fluid. This would suggest that vitreous sampling in such cases would be advocated.

In support of this, in a recent study by Singh et al. 57% of patients with a diagnosis of Eales disease had a demonstrable intraocular TB demonstrated by PCR testing of vitreous samples [27]. It is unclear whether similar results would be obtained if the same study have to be carried out in a population with a lower TB prevalence such as the UK. 

### 2.6. Other Intraocular Infections

Several other organisms may invade and infect the eye including fungi such as *Candida* and rarer bacterial infections, such as Whipples disease, Lyme disease, or *Bartonella* [28].

## 3. Suspected Intraocular Lymphoma

Intraocular lymphoma is an important masquerade of intermediate uveitis. In most cases, intraocular lymphoma involves the vitreous and the choroid and is a non-Hodgkins CD20+ B cell lymphoma, which is part of the spectrum of central nervous system (CNS) lymphoma. Approximately, 25% of patients with primary CNS lymphoma of this type develop intraocular involvement. Conversely, patients presenting with intraocular lymphoma have a high risk of developing CNS pathology, with over 50% developing disease [29].

Establishing a diagnosis of intraocular lymphoma is challenging and the gold standard requires demonstration of malignant cells or tissue. Often, a patient will have been treated with corticosteroids to address “uveitis.” This affects the yield and the phenotype of the cells in the vitreous. Ideally therefore, steroids should be rapidly tapered prior to vitreous sampling in order to increase the yield of lymphoma cells within the eye. A negative vitreous biopsy in the face of ongoing clinical suspicion is an indication for repeating a vitreous biopsy. Repeatedly negative sampling may necessitate a chorioretinal biopsy to be undertaken. Lymphoma cells are fragile and rapidly disintegrate, meaning that [30] obtaining an adequate vitreous sample requires special considerations and procedures [31]. Ideally, at least 2 mL of undiluted vitreous should be sampled, and the pathologist analysing the sample should be made aware to expect the sample and to analyse it, ideally within one hour of the procedure. If this is not possible, the specimen should be placed in a mild cytofixative, such as hepes-glutamic acid buffer mediated organic solvent protection effect (HOPE) or CytoLyt [32]. 

Features of lymphoma cells include atypical lymphoid cells with scant basophilic cytoplasm and a high nuclear : cytoplasmic ration and prominent nucleoli [33]. Haematoxylin and eosin staining can be used, however, Giemsa may be better at demonstrating the presence of lymphoma cells. As well as lymphoma cells, the vitreous may also contain inflammatory cells, fibrin, and cellular debris. Accurate diagnosis requires the skill of an experienced ocular pathologist [34].

Immunohistochemistry is used to stain for specific surface immune cell markers, including CD22, CD20, and CD19, thus further characterising the lymphoma cells. Germinal centre markers can also be identified, including CD10 [35].

Flow cytometry can also be employed in order to analyse the cells allowing characterisation of surface markers and surface antibodies. This technique also enables monoclonality to be demonstrated. There are several caveats to the use of flow cytometry; however, useful adjunctive information can be obtained by using the technique [7].

Analysis of the cytokines presented in the vitreous can be used as an adjunctive test in the diagnosis of lymphoma, and cytokine levels can be measured using enzyme immunoassay. Inflammatory cytokines, such as IL-6 or TNF-alpha, are found to be present in the eyes of patients with idiopathic inflammation [36]. This has also been demonstrated experimentally. Patients with lymphoma are found to have low levels of proinflammatory IL-6, but higher levels of the anti-inflammatory cytokine IL-10 are produced by B cells. The IL-10 : IL-6 ratio has been studied as a marker to support the presence of intraocular lymphoma, and it has been proposed that a ration greater that 1.0 is highly suggestive of intraocular lymphoma. 

It has also been proposed that the IL-10 level alone can also be used as a surrogate marker of lymphoma and can be obtained from an anterior chamber paracentesis with reportedly good sensitivity and specificity. In this study, the authors reported an aqueous level of 50 pg/mL to have a sensitivity and specificity of 0.89 and 0.93, respectively. Vitreous levels of 400 pg/mL yielded a specificity of 0.99 and a sensitivity of 0.8 [37].

Finally, monoclonality of B-cell populations is a feature of lymphoma and can be detected using molecular analysis. PCR is used to show rearrangements of the IgH gene, especially affecting the IgH variable region. Monoclonality of the more rare T-cell lymphomas can be demonstrated through the identification of TCR gene rearrangements [38].

In summary therefore, although cytology is the “gold standard” for diagnosing lymphoma, it is seen that the availability of these adjunctive techniques to test the vitreous can enhance the diagnosis especially when the laboratory technician is faced with a poor cellular yield from a vitreous sample. 

## 4. Vitreous Analysis in Patients with Autoinflammatory Uveitis

The majority of uveitis encountered in western uveitis clinics is diagnosed as being autoimmune or autoinflammatory. In approximately half of patients, intraocular inflammation occurs as part of a systemic disease, and intraocular findings may adhere to a characteristic phenotype [2].

Analysis of the vitreous in autoimmune or autoinflammatory uveitis has been undertaken mainly in a research setting. Animal models enable testing to be undertaken, whilst clinical studies offer an insight into the nature of the inflammatory environment.

Experimental models of uveitis support the proposal that inflammation occurs due to immune-mediated attack on retinal antigen [39]. Experimental autoimmune uveitis (EAU) is an immune-mediated response against soluble retinal antigens, found mainly around photoreceptor segments. T-cell-mediated attack against intraocular antigen is believed to be central in the mechanism of autoimmune uveitis. 

Following stimulation of T cells by antigen (which may be presented by antigen presenting cells in the eye), T cells differentiate into 3 main subtypes, which are characterised by the types of cytokines that they release. These subtypes include Th1, Th2, and Th17. In the context of EAU, T cells are polarised toward a Th1 response, whilst resolution of disease is associated with polarisation toward Th2 and regulatory T-cell phenotype. 

Ooi et al. reviewed the relevance of cytokines in both experimental autoimmune uveitis and also in patients affected with uveitis [36]. Proinflammatory cytokines are found to be present in patients and animal models of uveitis at high levels. These include Il1, IL2, IL6 IFNy, and TNFa.

TNF-alpha is a significant cytokine in autoimmune uveitis [40] and is the focus of targetted biologic therapy in the treatment of noninfectious uveitis [41]. It is released from monocytes, macrophages natural killer cells, and T cells and stimulated increased cellular infiltration by activating macrophages, increased in leukocytic infiltration and upregulating adhesion molecules [42]. Analysis of vitreous samples from animals models demonstrates high levels of TNF-a within the eye during inflammation.

 Studies examining the findings in the vitreous of patients with a prediagnosed condition have been undertaken. These have demonstrated different cytokine environments within the eyes of these patients, sometimes supporting the underlying diagnosis and enabling further understanding of the inflammatory process. Testing vitreous for cytokine levels is certainly not routine in clinical practice, and as seen from papers such as this, high levels of different inflammatory cytokines may occur with a range of inflammatory or infectious aetologies. 

In a paper by Nagata et al., the authors aim to report cytokines that are upregulated in the vitreous fluid of patients with ocular sarcoidosis to see whether a characteristic pattern can be observed [43]. They found that when levels of 27 different cytokines were measured, the vitreous levels of 17 cytokines were elevated in the patients with sarcoidosis compared with patients with idiopathic epiretinal membrane. As well as some cytokines being elevated, there were some that were lower in the patient group with sarcoidosis. The authors also correlated levels of inflammatory cytokines with the degree of cystoid macular oedema observed. 

## 5. Conclusion

Analysis of the vitreous is shown to be a valuable adjunct to the management of patients with intraocular inflammation [44]. Limitations of the tests are likely to become less as more advanced testing methods are introduced. The importance of selecting the appropriate tests to support a clinical suspicion is emphasised, as is the interpretation of test results within a clinical context.

## References

[1] Jabs DA, Nussenblatt RB, Rosenbaum JT (2005). Standardization of uveitis nomenclature for reporting clinical data. Results of the first international workshop. *American Journal of Ophthalmology*.

[2] Barisani-Asenbauer T, Maca S, Mejdoubi L, Emminger W, Machold K, Auer H (2012). Uveitis—a rare disease often associated with systemic diseases and infections—a systematic review of 2619 patients. *Orphanet Journal of Rare Diseases*.

[3] Rothova A, Ooijman F, Kerkhoff F, Van der Lelij A, Lokhorst HM (2001). Uveitis masquerade syndromes. *Ophthalmology*.

[4] Manku H, McCluskey P (2005). Diagnostic vitreous biopsy in patients with uveitis: a useful investigation?. *Clinical and Experimental Ophthalmology*.

[5] Rothova A, de Boer JH, ten Dam-van Loon NH (2008). Usefulness of aqueous humor analysis for the diagnosis of posterior uveitis. *Ophthalmology*.

[6] Trivedi D, Denniston AK, Murray PI (2011). Safety profile of anterior chamber paracentesis performed at the slit lamp. *Clinical and Experimental Ophthalmology*.

[7] Davis J, Miller D, Ruiz P (2005). Diagnostic testing of vitrectomy specimens. *American Journal of Ophthalmology*.

[8] Van Gelder RN (2001). Applications of the polymerase chain reaction to diagnosis of ophthalmic disease. *Survey of Ophthalmology*.

[9] Nandi K, Ranjan P, Therese L, Biswas J (2008). Polymerase chain reaction in intraocular inflammation. *The Open Ophthalmology Journal*.

[10] Yeung SN, Butler A, Mackenzie PJ (2009). Applications of the polymerase chain reaction in clinical ophthalmology. *Canadian Journal of Ophthalmology*.

[11] Muthiah MN, Michaelides M, Child CS, Mittchell SM (2007). Acute retinal necrosis: a national population-based study to assess the incidence, methods of diagnosis, treatment strategies and outcomes in the UK. *British Journal of Ophthalmology*.

[12] Davis JL (2012). Diagnostic dilemmas in retinitis and endophthalmitis. *Eye*.

[13] Tran THC, Rozenberg F, Cassoux N, Rao NA, LeHoang P, Bodaghi B (2003). Polymerase chain reaction analysis of aqueous humour samples in necrotising retinitis. *British Journal of Ophthalmology*.

[14] Balansard B, Bodaghi B, Cassoux N (2005). Necrotising retinopathies simulating acute retinal necrosis syndrome. *British Journal of Ophthalmology*.

[15] Harper TW, Miller D, Schiffman JC, Davis JL (2009). Polymerase chain reaction analysis of aqueous and vitreous specimens in the diagnosis of posterior segment infectious uveitis. *American Journal of Ophthalmology*.

[16] Cochrane TF, Silvestri G, McDowell C, Foot B, McAvoy CE (2012). Acute retinal necrosis in the United Kingdom: results of a prospective surveillance study. *Eye*.

[17] Safneck JR (2012). Endophthalmitis: a review of recent trends. *Saudi Journal of Ophthalmology*.

[18] Wittenberg LA, Maberley DA, Ma PE, Wade NK, Gill H, White VA (2008). Contribution of vitreous cytology to final clinical diagnosis fifteen-year review of vitreous cytology specimens from one institution. *Ophthalmology*.

[19] Joseph C, Lalitha P, Sivaraman K, Ramasamy K, Behera U (2012). Real-time polymerase chain reaction in the diagnosis of acute postoperative endophthalmitis. *American Journal of Ophthalmology*.

[20] Bispo PJM, de Melo GB, Hofling-Lima AL, Pignatari ACC (2011). Detection and gram discrimination of bacterial pathogens from aqueous and vitreous humor using real-time PCR assays. *Investigative Ophthalmology and Visual Science*.

[21] Schiedler V, Scott IU, Flynn HW, Davis JL, Benz MS, Miller D (2004). Culture-proven endogenous endophthalmitis: clinical features and visual acuity outcomes. *American Journal of Ophthalmology*.

[22] Meisler DM, Palestine AG, Vastine DW (1986). Chronic Propionibacterium endophthalmitis after extracapsular cataract extraction and intraocular lens implantation. *American Journal of Ophthalmology*.

[23] Bodaghi B, Touitou V, Fardeau C, Paris L, LeHoang P (2012). Toxoplasmosis: new challenges for an old disease. *Eye*.

[24] Montoya JG, Parmley S, Liesenfeld O, Jaffe GJ, Remington JS (1999). Use of the polymerase chain reaction for diagnosis of ocular toxoplasmosis. *Ophthalmology*.

[25] De Groot-Mijnes JDF, Rothova A, Van Loon AM (2006). Polymerase chain reaction and goldmann-witmer coefficient analysis are complimentary for the diagnosis of infectious uveitis. *American Journal of Ophthalmology*.

[26] Fardeau C, Romand S, Rao NA (2002). Diagnosis of toxoplasmic retinochoroiditis with atypical clinical features. *American Journal of Ophthalmology*.

[27] Singh R, Toor P, Parchand S, Sharma K, Gupta V, Gupta A (2012). Quantitative polymerase chain reaction for Mycobacterium tuberculosis in so-called Eales' disease. *Ocular Immunology and Inflammation*.

[28] Touitou V, Fenollar F, Cassoux N (2012). Ocular whipple's disease: therapeutic strategy and long-term follow-up. *Ophthalmology*.

[29] Péer J, Hochberg FH, Foster CS (2009). Clinical review: treatment of vitreoretinal lymphoma. *Ocular Immunology and Inflammation*.

[30] Coupland SE (2008). The pathologist's perspective on vitreous opacities. *Eye*.

[31] Karma A, von Willebrand EO, Tommila PV, Paetau AE, Oskala PS, Immonen IJ (2007). Primary intraocular lymphoma: improving the diagnostic procedure. *Ophthalmology*.

[32] Coupland SE, Perez-Canto A, Hummel M, Stein H, Heimann H (2005). Assessment of HOPE fixation in vitrectomy specimens in patients with chronic bilateral uveitis (masquerade syndrome). *Graefe’s Archive for Clinical and Experimental Ophthalmology*.

[33] Coupland SE, Damato B (2008). Understanding intraocular lymphomas. *Clinical and Experimental Ophthalmology*.

[34] Sen HN, Bodaghi B, Hoang PL, Nussenblatt R (2009). Primary intraocular lymphoma: diagnosis and differential diagnosis. *Ocular Immunology and Inflammation*.

[35] Raparia K, Chang CC, Chévez-Barrios P (2009). Intraocular lymphoma: diagnostic approach and immunophenotypic findings in vitrectomy specimens. *Archives of Pathology and Laboratory Medicine*.

[36] Ooi KGJ, Galatowicz G, Calder VL, Lightman SL (2006). Cytokines and chemokines in uveitis—is there a correlation with clinical phenotype?. *Clinical Medicine and Research*.

[37] Cassoux N, Giron A, Bodaghi B (2007). IL-10 measurement in aqueous humor for screening patients with suspicion of primary intraocular lymphoma. *Investigative Ophthalmology and Visual Science*.

[38] Baehring JM, Androudi S, Longtine JJ (2005). Analysis of clonal immunoglobulin heavy chain rearrangements in ocular lymphoma. *Cancer*.

[39] Caspi RR, Silver PB, Luger D (2008). Mouse models of experimental autoimmune uveitis. *Ophthalmic Research*.

[40] Dick AD, McMenamin PG, Körner H (1996). Inhibition of tumor necrosis factor activity minimizes target organ damage in experimental autoimmune uveoretinitis despite quantitatively normal activated T cell traffic to the retina. *European Journal of Immunology*.

[41] Suhler EB, Smith JR, Wertheim MS (2005). A prospective trial of infliximab therapy for refractory uveitis: preliminary safety and efficacy outcomes. *Archives of Ophthalmology*.

[42] Dick AD, Forrester JV, Liversidge J, Cope AP (2004). The role of tumour necrosis factor (TNF-*α*) in experimental autoimmune uveoretinitis (EAU). *Progress in Retinal and Eye Research*.

[43] Nagata K KS, Maruyama K, Uno K (2012). Simultaneous analysis of multiple cytokines in the vitreous of patients with sarcoid uveitis. *Investigative Ophthalmology & Visual Science*.

[44] Lobo A, Lightman S (2003). Vitreous aspiration needle tap in the diagnosis of intraocular inflammation. *Ophthalmology*.

